# Clinical referral to the NHS following multi-cancer early detection test results from the NHS-Galleri trial

**DOI:** 10.3389/fonc.2025.1511816

**Published:** 2025-05-08

**Authors:** Ian Lowenhoff, Saoirse Dolly, Rebecca Dowinton Smith, Sara Hiom, Liz Holmes, Peter Johnson, Laura King, Richard D. Neal, Thomas Round, Charles Swanton, Lennard Y. W. Lee

**Affiliations:** ^1^ Cancer Research UK Cancer Prevention Trials Unit, Queen Mary University of London, London, United Kingdom; ^2^ Guy’s and St Thomas’ National Health Service (NHS) Foundation Trust, London, United Kingdom; ^3^ GRAIL Bio UK, Ltd., London, United Kingdom; ^4^ NHS England, London, United Kingdom, London, United Kingdom; ^5^ Department of Health and Community Sciences, University of Exeter, Exeter, United Kingdom; ^6^ Department of Population Health Sciences, King’s College London, London, United Kingdom; ^7^ UCL Cancer Institute, University College London (UCL), London, United Kingdom; ^8^ The Francis Crick Institute, London, United Kingdom; ^9^ Nuffield Department of Medicine, University of Oxford, Oxford, United Kingdom

**Keywords:** multi-cancer early detection, liquid biopsy, cell-free nucleic acids, population screening, randomised controlled trial, secondary care, urgent referral, health care delivery

## Abstract

**Introduction:**

The large, randomised, controlled NHS-Galleri trial (NCT05611632) is assessing the clinical utility of a multi-cancer early detection (MCED) test for asymptomatic cancer screening in England. We describe how we enabled the efficient referral of trial participants into existing National Health Service (NHS) urgent suspected cancer pathways for diagnostic investigations.

**Methods/Results:**

Participants were enrolled across eight of the 21 Cancer Alliance regions in England, served by 56 Hospital Trusts. We used the existing NHS e-Referral Service (e-RS) and a new e-referral form to enable referrals from the trial into any participating Trust, and to standardise information provided with trial participant referrals. Referrals were made by trial nurses directly into secondary care, minimising any additional burden on primary care. At most Trusts, a designated Trust-based referral coordinator triaged referrals and referred participants into the most appropriate local pathway, selected based on the tissue type or organ associated with the cancer signal (cancer signal origin; CSO). At other Trusts, trial nurses referred participants into the appropriate pathway. Guidance mapping predicted CSO(s) to NHS pathways was provided by the trial team to help clinicians understand trial referrals. The trial team and Trust referral coordinators were responsible for central and Trust-level safety netting measures, respectively.

**Conclusions:**

To our knowledge, the NHS-Galleri trial has established the first model for the standardised clinical referral of asymptomatic individuals from a trial into NHS standard-of-care cancer pathways. We hope insights from our work could help accelerate screening trial conduct in the UK, and support MCED population screening programme implementation in future.

## 1 Introduction

Prognosis, treatment options, quality of life and survival are generally better for individuals with cancer who are diagnosed at early (I or II) versus late stages (III or IV) (1, 2). Existing individual cancer screening programmes account for approximately 6% of all cancers detected in England each year (3); these cancers are more likely to be detected at an early stage than those not detected by screening (4). The percentage of cancers diagnosed early in England has remained relatively low, at approximately 54% since 2013 (5); innovative approaches are required to improve this situation. Multi-cancer early detection (MCED) tests are designed to detect clinically significant cancers in multiple organ systems before symptomatic presentation and detection in usual care (6). MCED tests could increase the proportion of cancers diagnosed early, provided they are integrated effectively into existing healthcare systems.

One blood-based MCED test (Galleri^®^; GRAIL, Inc., Menlo Park, California, USA) analyses methylation patterns on cell-free DNA. Detection of a methylation pattern associated with cancer is returned as a cancer signal detected (CSD) test result. When a cancer signal is detected, further analysis is performed to predict the tissue type or organ associated with the cancer signal (cancer signal origin; CSO). (7). The clinical utility of this MCED test for asymptomatic population screening in combination with existing NHS cancer screening programmes is being assessed in England among individuals aged 50–79 years in the large, randomised, controlled NHS-Galleri trial (NCT05611632) (6). Participants in the NHS-Galleri finished their third of three appointments in summer 2024, completing three rounds of annual screening. Outcomes data will be collected until summer 2025, and final trial results are expected in 2026.

Participants in the NHS-Galleri trial who received a CSD test result were recommended to be referred for diagnostic investigation to confirm whether cancer is present. This is the same for individuals in England who receive a single-cancer screening result indicating suspected cancer. However, there is little data on referral pathways and diagnostic investigation following an MCED test result indicating suspected cancer, and the data are mostly limited to a US setting, with no consensus on best practice (7–11). The NHS-Galleri trial represented an opportunity to establish how individuals with a CSD result that includes a predicted CSO could be efficiently referred for diagnostic investigation in a national healthcare system (6), whilst maintaining as near equivalent diagnostic investigation as possible between the intervention and control arms.

Here, we describe how existing NHS standard-of-care cancer pathways were used for the referral and diagnostic investigation of participants in the NHS-Galleri trial. We describe the challenges and adaptations used, and highlight elements of this approach that could usefully inform screening trial conduct in the UK and potential implementation of MCED tests for national-level population screening in future.

## 2 Material and methods

### 2.1 Referring participants from the trial into the NHS

**Figure 1:**
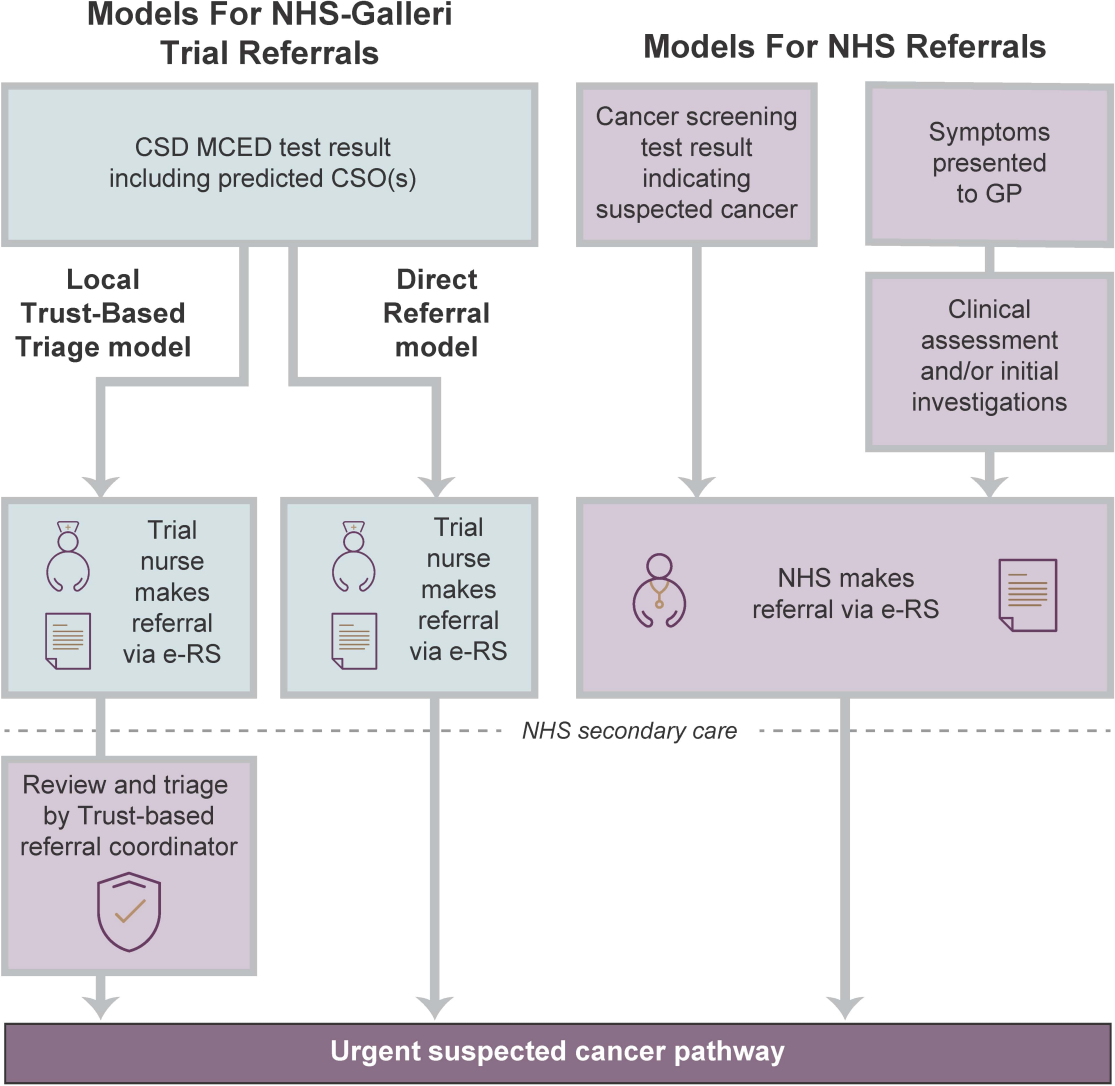
Referral Models Used in the NHS-Galleri Trial Compared With Standard NHS Referral. In the NHS-Galleri trial, two models were used for referral of participants to secondary care: Local Trust-Based Triage and Direct Referral. In the Local Trust-Based Triage model, referrals were reviewed and triaged by a designated Trust-based referral coordinator, who then re-routed referrals into the appropriate standard-of-care urgent suspected cancer pathway. In the Direct Referral model, trial nurses made referrals directly into the relevant urgent suspected cancer pathway. CSD, cancer signal detected; CSO, cancer signal origin; e-RS, e-referral system; GP, general practitioner; MCED, multi-cancer early detection; NHS, National Health Service.

Referral pathways for suspected cancer can be complex to navigate (17), but current systems contribute to improved patient outcomes (18) and are well positioned to support referrals based on certain CSD test results. The CSOs predicted by MCED tests are a novel type of clinical indicator for the referral of asymptomatic individuals into existing NHS urgent suspected cancer pathways. The use of CSOs minimises downstream adaptations to accommodate this new technology into standard-of-care procedures (**Figure 1**). Adaptations were primarily required to accommodate the initiation of trial referrals into each of the 56 participating local NHS Trusts (which serve eight of 21 English Cancer Alliances) by the trial nurses via the existing NHS e-Referral Service (e-RS). Requirements of the process were to: [1] enable efficient referral of trial participants into NHS Hospital Trusts in England, which provide secondary care services including cancer diagnostic testing; [2] facilitate the receipt of trial participants by Trusts; and [3] support clinical decision making on participant diagnostic journeys to reinforce standard-of-care approaches. Overall, these adaptations formed a novel interface between the NHS-Galleri trial and Trusts to facilitate centralised referrals from a clinical trial into the NHS.

We identified and addressed clinician and systems-level challenges in our approach, including: clinician understanding of the MCED test, interpretation of results, and appropriate referral pathway decisions based on CSD test results; establishing a novel point of entry into the existing urgent suspected cancer pathways; and ensuring follow-up and safety netting procedures, especially in more complex cases (e.g., those with two predicted CSOs). The trial nurses managed potential anxiety around the CSD test result and supported adequate understanding of CSD test result implications among participants in the trial. This included that it is not a cancer diagnosis, therefore follow-up diagnostic testing is required to confirm cancer; that false positive results can occur; and that results are not predictive of future genetic cancer risk. The psychological impact of having a CSD result following a blood-based MCED test is currently being explored in another study (19).

In this section, we describe how we developed adaptations to support optimal referral of NHS-Galleri trial participants following a CSD test result, and the benefits and limitations of these adaptations.

### 2.2 Optimising referral of trial participants into secondary care

We developed a national, standardised e-referral form to facilitate and expedite referrals of trial participants from a centralised hub via the existing e-RS to a number of pre-specified Trusts in England for diagnostic testing. The form sent with the referral included the MCED test result report, which could include one or two predicted CSOs. The trial e-referral form differed from standard NHS referral forms in that it did not include additional healthcare information (e.g., medical history, blood results or a Summary Care Record), because the trial nurses did not have access to NHS clinical data.

Trial nurses were NHS employees, so they could access and make referrals from the trial via the existing NHS e-RS. NHS Trusts could contact trial nurses using the contact details on the e-referral form to request support with understanding referrals. Trial nurses also delivered CSD test results to participants by telephone, using a script (followed by a letter to the participant and their GP). This was to ensure key checks were undertaken and specific points were communicated, ensuring participants with a CSD result received sufficient information in a standardised way and adequately understood their result.

There were several benefits to this approach to referral from the trial. First, the e-RS is an existing system, accessible nationally to both the trial nurses and secondary care. Second, this streamlined, digital pathway allowed the transfer of information electronically, without the need for physical referral forms, reducing the likelihood of referrals getting lost and the risk of data errors. Third, use of the e-RS permitted monitoring of referral acceptances by Trusts, which was an important safety netting measure to ensure follow-up for all trial participants. Finally, referral of trial participants did not place additional burden on primary care, because whilst GPs were notified if a patient of theirs had a CSD result, trial nurses were responsible for referring participants directly into secondary care.

Appending health information to referrals from the trial was not considered feasible, advisable (due to increased risk of exposing patient health information), or necessary. Patient clinical information had to be accessed/collected in secondary care, creating some additional burden compared with standard referrals. However, Summary Care Records could be accessed via NHS Spine (**Table 1**) (14) or through local secondary care electronic patient record systems The NHS referral system is largely standardised, and uses shared software across Trusts. However, a small number of Trusts requested email referrals instead of setting up or using a single e-RS referral service address; these requests were facilitated by the trial team.

**Table 1:** Glossary of key terms used in this paper and their definitions.

Term used	Definition
Cancer Alliance	An area-based NHS organisational unit that coordinates cancer care and aims to improve outcomes for patients locally.
Cancer signal detected (CSD)	An MCED test result indicating the detection of a methylation profile in the cell-free DNA fraction commonly associated with cancer. This is not a cancer diagnosis, and requires further diagnostic testing to confirm or rule out a cancer diagnosis.
Cancer signal origin (CSO)	A prediction that offers information about the tissue type or organ associated with the cancer signal in the case of a CSD test result; it can be used to guide downstream diagnostic testing. There may be one or two CSOs listed in the test result report.
Clinical champion	Local-level point of contact for clinicians who receive participants referred from the NHS-Galleri trial. This individual is equipped with a good understanding of the trial, responds to queries from clinicians in their Trust, and acts as a point for escalation if a Trust declines a referral. All clinical champions are cancer clinicians (nurses or doctors).
Faster diagnosis standard	A standard introduced by the NHS to ensure patients who are referred for suspected cancer receive a timely diagnosis. This standard includes a target to diagnose or rule out cancer within 28 days of referral by a GP or a cancer screening programme (12). This replaced the two-week wait (2ww) target, which was in place at the start of the NHS-Galleri trial, and aimed for those with suspected cancer to see a specialist within 14 days of referral.
General practitioner (GP)	Family physician in the UK.
Multi-cancer early detection (MCED) test	A test designed to identify clinically significant cancers in multiple organ systems before symptomatic presentation and detection in usual care (6).
National Health Service (NHS)	The publicly funded healthcare system in England.
NHS England	The commissioning body for healthcare in England.
NHS e-Referral Service (e-RS)	An online NHS system used to make and manage referral bookings, originally designed for GPs to refer patients to secondary care services (13).
NHS Hospital Trust	An organisational unit in the NHS that provides secondary care services, including cancer diagnostic testing.
NHS Spine	An inter-organisational online platform that supports the IT infrastructure for health and social care in England (14).
National Institute for Health and Care Excellence (NICE)	A non-departmental public body that provides evidence-based guidance for health and care professionals in England.
Nonspecific symptoms (NSS) pathway	A diagnostic pathway for patients with non-site-specific symptoms that could indicate cancer; diagnostic testing via this pathway may take place in an RDC, where available.
Patient administrator	An individual who supports the efficient running of a clinical team by liaising with patients and healthcare professionals.
Patient navigator	An individual who facilitates patients’ journeys through the NHS, providing advocacy, information and assistance to patients.
Rapid Diagnostic Centre (RDC)	A single point of access to a diagnostic pathway for patients with non-site-specific symptoms that could indicate cancer (15); RDCs are currently being rolled out across England, and are not yet available in all locations.
Trial nurse	An NHS-employed nurse who delivers the CSD test result to participants in the NHS-Galleri trial, and initiates referrals of trial participants with a CSD test result into secondary care for diagnostic testing. Trial nurses also support Trusts to act on referrals they receive.
Trust referral coordinator	Named individual(s) who act as a central point of contact at NHS Trusts and are responsible for the tracking and safety-netting of referrals received from the trial. They may subsequently refer participants into the appropriate urgent suspected cancer pathway based on the predicted CSO(s) in the test result report. Trust referral coordinators may be administrators or healthcare professionals.
Urgent suspected cancer pathway	A standard-of-care diagnostic referral pathway in the NHS for the referral of individuals with suspected cancer symptoms or a single-cancer screening test result indicating suspected cancer. Of these, 11 pathways are organised by anatomical site, and there is an additional NSS pathway (16).

### 2.3 Facilitating the receipt of trial participants by NHS Trusts

#### 2.3.1 Referral models

In collaboration with Cheshire and Merseyside and South East London Cancer Alliances, we developed and tested two models for referral of participants with a CSD test result into standard-of-care cancer pathways, which are both currently being used in the trial (**Figure 1**).

In the Local Trust-Based Triage model, a designated Trust-based referral coordinator triaged referrals and referred participants into the most appropriate local urgent suspected cancer pathway. This became the preferred model for the trial. In this model, trial nurses referred participants to NHS secondary care via the e-RS, where the referral was reviewed, triaged, and added to the local monitoring system (e.g., Somerset Cancer Register) by a designated Trust-based referral coordinator. The referral coordinator then re-routed referrals into the appropriate standard-of-care urgent suspected cancer pathway based on the predicted CSO(s) in the test result report, or to an NSS pathway or RDC (**Table 1**). If no NSS pathway or RDC was available, the trial team helped Trusts using this model to set up a dedicated e-RS pathway that could be used solely for NHS-Galleri trial referrals. Two Trusts adopted a Local Trust-Based Triage model in which a designated Pre-Diagnosis Service Team contacted the patient, sent the referral to a central cancer hub where the referral was registered, and progressed the patient through the relevant urgent suspected cancer pathway(s). The Pre-Diagnosis Service Team attended patient tracking meetings, and assisted with delays in the diagnostic journey.

One benefit of the Local Trust-Based Triage model was that referral coordinators had local operational knowledge of the Trust, and were thus well placed to triage referrals and refer participants into the most appropriate pathway in their locality. This model lent itself well to a centralised referral process. Another benefit was that referral coordinators were able to monitor each participant’s diagnostic journey, in collaboration with the clinical champion. This was particularly important for cases that required referral into more than one pathway for a conclusive diagnosis; sequential referrals could add to the time it took to complete the diagnostic journey.

In the Direct Referral model, trial nurses made referrals directly into the relevant urgent suspected cancer pathway, based on the predicted CSO(s) in the test result report (**Figure 1**). The referral was also subsequently added to the local monitoring system. The main benefit of this model was that it placed no additional burden on Trusts to decide which urgent suspected cancer pathway was most appropriate for each participant. However, trial nurses making these decisions had less local operational knowledge than a Trust referral coordinator and often faced complexity in deciding between local pathway options. Although supporting information about the trial was sent with each referral, there was also an increased risk of the referral being erroneously declined. This was likely due to a lack of understanding about the trial process, given each secondary care clinician in participating trusts may have received only a small number of referrals from the trial, if any. For example, clinicians may have expected that referrals would come with results from initial investigations, usually completed in primary care, that are part of standard referrals (e.g., faecal immunochemical testing for the bowel cancer pathway). In addition, in cases where two possible CSOs are given, there was also a risk that the second predicted CSO may have not been investigated, because cancer type-specialist clinicians may have been unaware of the trial.

The choice of model(s) used by each Trust was based primarily on the feasibility of setting up the recommended Local Trust-Based Triage model, but may have been influenced by systems-related factors, e.g., if specialist diagnostic services are provided by another care provider, who may have a preference about how they receive patient referrals.

A small number of Trusts used a hybrid model, in which most referrals were made via Local Trust-Based Triage, but Direct Referral was used for some specialties, with these referrals sometimes crossing Cancer Alliance borders.

**Figure 2:**
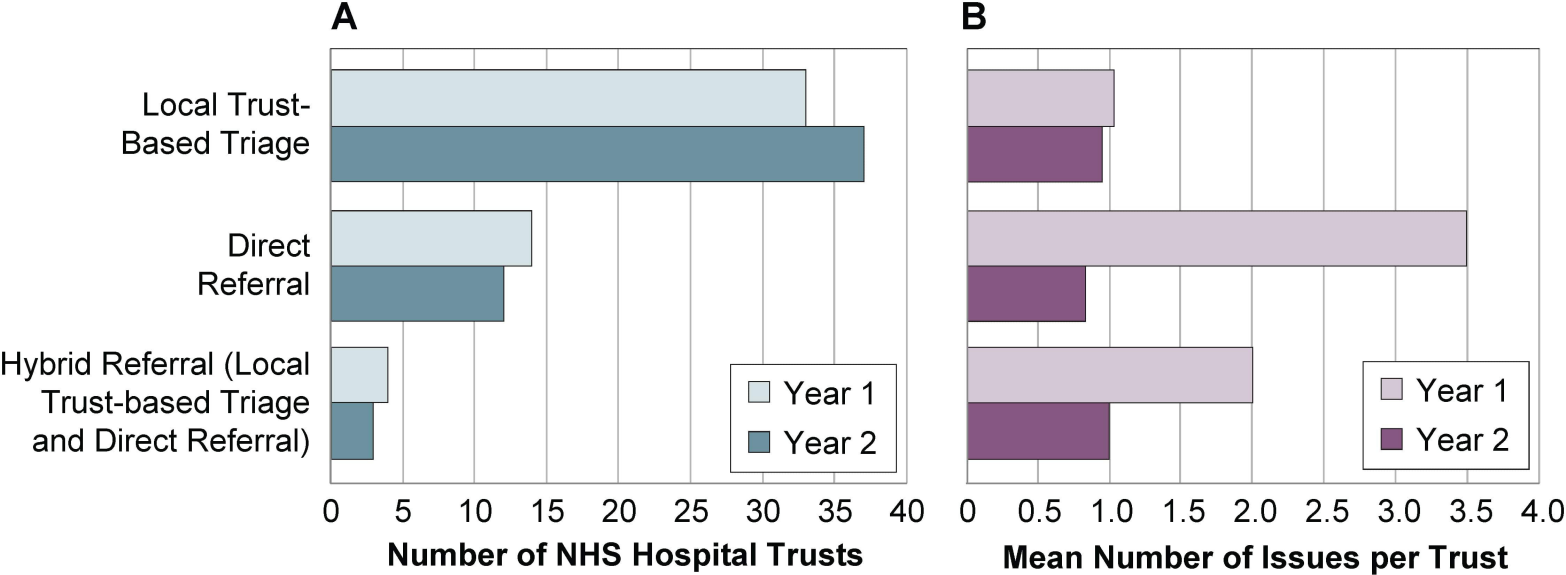
Number of **(A)** NHS Hospital Trusts Using Local Trust-Based Triage, Direct Referral, and Hybrid (Local Trust-Based Triage and Direct Referral) Models and **(B)** Mean Number of Issues Per Trust Using Local Trust-Based Triage, Direct Referral, and Hybrid Models in Year One and Year Two of the NHS-Galleri Trial. Year one: N = 51 Trusts; year two: N = 52 Trusts. Data were collected up to 4th December 2023.

At the end of the first year of the trial, 64.7% of Trusts were using the recommended Local Trust-Based Triage model, which increased to 71.2% of Trusts by the end of the second year (**Figure 2A**). There were approximately three times more referral issues (i.e., referrals not managed according to current clinical guidance) per Trust raised among Trusts using Direct Referral in the first year compared with those using the Local Trust-Based Triage model (**Figure 2B**). A major source of referral issues was confirmed as a lack of awareness of the trial among clinicians at the Trusts. Far fewer issues were raised with Local Trust-Based Triage than Direct Referral, because the referral coordinator in the Local Trust-Based Triage model has operational knowledge of both the trial and the Trust, supporting appropriate management of referrals.

Several Trusts switched to Local Trust-Based Triage due to issues they experienced with Direct Referral in the first year.

The mean number of issues attributable to the Direct Referral model decreased substantially by the end of the second year of the trial (**Figure 2B**) as fewer Trusts used this model in the second year. The mean number of issues reported by Trusts using Local Trust-Based Triage decreased only slightly (**Figure 2B**), with most issues likely due to the increased number of Trusts using this model, and a lack of familiarity among those who switched to this model in the second year.

#### 2.3.2 Safety netting

In the current NHS system, the healthcare professional who makes an urgent suspected cancer referral, usually a GP, actively ensures referrals for their patients are followed up. Their practice team makes an urgent diagnostic appointment, and ensures patients have received their appointment (16).

Given that referrals from the NHS-Galleri trial bypassed primary care, the trial team was responsible for central safety netting measures, including contacting participants to ensure appointments were set up and supporting participants to understand the referral and diagnostic process. Trial nurses sent reconciliation emails to each Trust to ensure that the number of referrals sent matched the number received by the Trust, and check whether referrals were made by reviewing actions recorded in the e-RS.

During the initial call in which trial nurses delivered a CSD test result, participants were briefed about what to expect once they were referred into the NHS. The trial team provided all participants with information on how to make contact if they had any concerns following referral into the NHS. Where possible, participants were provided with local cancer team contacts to facilitate appointment follow-up. The trial team contacted participants approximately every seven days after referral to ensure that the participant was offered an appointment for clinical assessment; if an appointment was not secured, the trial team escalated to the trial coordinator at the relevant Trust. During this check-in call, participants could also raise any concerns they had, and the trial team could provide support if it was required.

Named trial clinical champions were responsible for local safety netting at each Trust. Their responsibilities included responding to any simple queries from receiving clinicians, and escalating questions relating to the referral to the trial team. Trust referral coordinators, patient administrators, and patient navigators (**Table 1**) also supported the safety netting process. This included tracking the receipt and management of referrals in accordance with National Institute for Health and Care Excellence (NICE) Suspected Cancer: Recognition and Referral guidelines (16) as outlined in the trial referral guidance document (see section titled: *Guidance document to support optimal clinical decision making*).

To facilitate this process, trial clinical champions and Trust referral coordinators were established at each Trust, and the relevant service names, clinics, and urgent suspected cancer pathway e-RS codes were identified.

### 2.4 Supporting clinical decision making on participant diagnostic journeys

Any diagnostic investigation for trial participants takes place within the NHS, and is considered to be outside of the trial. Receiving Trusts are responsible for the clinical management of referred trial participants, and diagnostic investigation is conducted according to national (16) and local guidance for urgent suspected cancer referrals, following clinical assessment. This ensures that diagnostic journeys for trial participants in the intervention arm were generally managed according to systemised, standard-of-care practices.

#### 2.4.1 Cancer signal origin mapping to urgent suspected cancer pathways

We broadly matched all CSO options resulting from a CSD test result to existing adult NHS urgent suspected cancer pathways (**Table 2**), using advice and clinical expertise from a clinical advisory group consisting of primary and secondary healthcare professionals external to the trial. The clinical advisory group resolved situations in which it was challenging to map CSOs to existing urgent suspected cancer pathways, e.g., for the ‘bone and soft tissue’ CSO, which does not differentiate between these two cancer types.

**Table 2:** Map of cancer signal origins (CSOs) to standard-of-care urgent suspected cancer pathways described in National Institute for Health and Care Excellence (NICE) guidance (16).

CSO(s)	Urgent Suspected Cancer Pathway
Anus; Colon, Rectum	Lower gastrointestinal
Bladder, Urothelial Tract; Kidney; Prostate	Urology
Bone and Soft Tissue	Sarcoma
Breast	Breast
Cervix; Ovary; Uterus	Gynaecological
Head and Neck; Thyroid Gland	Head and neck
Liver, Bile Duct; Pancreas, Gallbladder; Stomach, Oesophagus	Upper gastrointestinal
Lung	Lung
Lymphoid Lineage; Myeloid Lineage; Plasma Cell Lineage	Haematology
Melanocytic Lineage	Skin
Neuroendocrine Cells of Lung or Other Organs	Lung/Other A referral may be made to a local neuroendocrine multidisciplinary Trust, if available

Where more than one CSO is present, Trusts may choose to refer participants using a nonspecific symptoms (NSS) pathway or a Rapid Diagnostic Centre (RDC), where available.

This map was intended to help trial nurses and Trust referral coordinators refer participants to the most appropriate urgent suspected cancer pathway in the absence of any symptoms. For Local Trust-Based Triage, this required Trusts to set up their referral pathways on the e-RS to ensure participants could be referred from secondary care into urgent suspected cancer pathways corresponding to their predicted CSO(s).

The downstream diagnostic pathway for referrals from the trial were thus the same as for referrals from standard points of entry in the NHS. This adaptation minimised deviation from standard-of-care practices in the intervention arm of the trial, and additional burden on secondary care. Although some urgent suspected cancer pathways go straight to a diagnostic test in usual care, trial participants were referred without accompanying medical history, so initial clinical assessment was advised prior to formulating a diagnostic plan, as outlined in the trial guidance document (see section titled: *Guidance document to support optimal clinical decision making*). This is aligned with NICE guidance, where clinicians are advised to undertake additional clinical assessments where indicated (16).

The national target in England for urgent suspected cancer referrals via standard points of entry was also applied to referrals from the trial, to ensure urgency and timeliness in the diagnostic investigation of referrals. During the first two years of the trial, the target was to offer an appointment in secondary care within two weeks of referral (two-week wait [2ww]). Following an update of the national target in October 2023, the target became to confirm or rule out a cancer diagnosis within 28 days of referral (12, 16).

#### 2.4.2 Guidance document to support optimal clinical decision making

We developed a guidance document for clinicians to support the interpretation of existing standard-of-care practice following a CSD test result during the NHS-Galleri trial. The document did not introduce any new clinical guidelines; rather, it emphasised the use of existing national and local guidelines, clinical judgement, and shared clinician–patient decision making. This included guidance in cases where no cancer diagnosis was confirmed following diagnostic investigation of the CSO(s) listed; this was because, due to the high positive predictive value of the MCED test, these patients have a greater residual cancer risk than the NICE-recommended cancer risk threshold that warrants diagnostic investigation in symptomatic individuals (16, 20). The document also encouraged incorporation of participant-specific factors, such as participant overall fitness and the likelihood that they will benefit from further investigation or treatment, into clinical decision making. It also included an extensive frequently asked questions section on managing referrals and the trial in general. An additional aim of the document was to reassure clinicians that current standard-of-care guidance could be followed to ensure adequate diagnostic investigation for participants referred from the trial.

Regular (weekly at first, then fortnightly) clinical meetings were held between representatives from the trial team and NHS England to oversee, review, and advise on any issues regarding referrals and the interface between the trial and Trusts, review clinician feedback on the guidance document, and consider improvements to the information provided. The document was thus updated frequently to capture key learnings and safety information from Trust experiences of managing referrals from the trial. Some examples include how to manage cases in which trial participants had already been diagnosed or referred for cancer, or in which two predicted CSOs were present in the MCED test result report. There was also regular clinical engagement with Trusts, e.g., via webinars. This ensured that best practice was shared as quickly as possible. This likely contributed to the overall reduction in referral issues raised during the trial.

## 3 Discussion

We utilised existing NHS e-RS systems to enable the referral of participants with an MCED test result indicating suspected cancer from a large, randomised trial into the NHS using light-touch adaptations. Referral for trial participants with CSD test results followed existing pathways that healthcare professionals working in NHS Hospital Trusts frequently use and understand well. The novel components of the referral process were to enable referral from a trial into the NHS using an existing electronic system, and refer individuals with no cancer symptoms into symptomatic pathways. This process was designed to be simple and low-burden for secondary care.

Crucially, this referral process placed no additional direct workload burden on primary care. However, as GPs were not involved in referral or diagnostic investigation of trial participants, there were occasionally challenges if secondary care clinicians rejected referrals or redirected participants to their GP due to their expectation that recommended tests should have been done at the point of referral. Issues such as these were discussed and resolved in clinical meetings between NHS England and the trial team.

Our approach demonstrates that trial participants could successfully be referred into standard diagnostic pathways in the NHS following a CSD test result, whilst maintaining as near equivalent diagnostic investigation as possible between the intervention and control arms. Participants were expected to receive diagnostic investigation broadly in line with the current standard-of-care and national targets in England (12, 16). Diagnostic evaluation was supported by the use of existing urgent suspected cancer pathways, and the freedom of clinicians to use their clinical judgement and incorporate individual patients’ needs and wishes when making decisions in accordance with national and local guidelines. In addition, safety netting measures both within each participating Trust and by the trial team helped to ensure all referrals were carried forward, and participant diagnostic journeys adequately resolved. Participants were reminded of the importance of attending routine screening programmes and symptom vigilance, irrespective of the outcome of their referral.

One challenge of our approach was that the setup work required to implement some of the new features was not straightforward. This includes explicitly establishing Trust referral coordinators, clinical champions, service names, clinics, and e-RS codes at each Trust. Another challenge was raising and maintaining levels of awareness of the trial among the thousands of clinicians at the participating Trusts. We took a practical approach to ensure that receiving clinicians have the information they need to adequately manage referrals from the trial by providing summary information about the trial on the national referral form, and establishing clinical champions at each Trust who acted as the point of contact for clinicians. Our trial guidance document, issued to clinicians, was updated regularly to incorporate best practice learning during the trial. We worked closely with the eight participating Cancer Alliances to disseminate key information to clinicians and administrators, facilitating the effective operation of referral pathways. The trial team also provided intranet toolkits and briefing documents for primary and secondary care that Trusts could disseminate using their own internal communications channels. Finally, we hosted webinars and question-and-answer sessions to help clinicians understand the trial referral process.

This innovative approach facilitated referral of participants from the NHS-Galleri trial into the NHS for diagnostic evaluations. Insights from our work may help to accelerate the conduct of future screening trials in the UK, and inform any potential future implementation of MCED tests for population screening, should clinical utility be demonstrated. They could also provide transferable knowledge to expedite access to other novel healthcare technologies. Early evidence suggests routing referrals through a single point of entry at the receiving Trust appears to be the optimal route for diagnostic investigation following a CSD test result (8).

There are limitations to the extent to which this trial-based process could be applied in other settings, particularly the implementation of a national-level population screening programme in the NHS. In addition, the practicalities of how this approach might be adopted by the NHS could influence its effectiveness. For example, integrating a new e-referral form into an existing e-RS will require some setup time, and Trusts may opt to handle referrals differently. There may be other, as-yet unknown challenges related to the applicability and scalability of our approach. Similar challenges are likely to apply to any novel intervention or approach that must integrate into the health service.

### 3.1 Conclusions

We used a novel approach to enable referral of participants from a large randomised controlled trial into appropriate existing urgent suspected cancer pathways across Cancer Alliances and NHS Hospital Trusts in England. This approach maximised the probability that participants with a test result indicating suspected cancer received a standard-of-care diagnostic investigation within the NHS. Insights from our work could help accelerate the conduct of other large screening trials in the UK, and support the implementation of national population screening programmes in future.

## Data Availability

The original contributions presented in the study are included in the article/supplementary material. Further inquiries can be directed to the corresponding author.
